# Draft Genome Sequence of *Methyloligella halotolerans* С2^T^, a New Halotolerant Methylotroph, Accumulating Poly-3-Hydroxybutyrate and Ectoine

**DOI:** 10.1128/genomeA.01189-16

**Published:** 2016-10-27

**Authors:** Oleg V. Vasilenko, Nina V. Doronina, Maria N. Shmareva, Sergey V. Tarlachkov, Yuri A. Trotsenko

**Affiliations:** a G.K. Skryabin Institute of Biochemistry and Physiology of Microorganisms, Russian Academy of Sciences, Pushchino, Moscow Region, Russia; b Branch of Shemyakin and Ovchinnikov Institute of Bioorganic Chemistry, Russian Academy of Sciences, Pushchino, Moscow Region, Russia

## Abstract

*Methyloligella halotolerans* С2^T^ is a moderate halophilic obligate methylotroph, accumulating ultra-high-molecular-weight poly-3-hydroxybutyrate (up to 8 to 10 MDa) from methanol. Here we report a draft genome and annotation of *Methyloligella halotolerans* C2^T^ (VKM B-2706^T^ = CCUG 61687^T^ = DSM 25045^T^).

## GENOME ANNOUNCEMENT

The genus *Methyloligella* currently includes two species with validly published names, *M. solikamskensis* SK12^T^ and *M. halotolerans* С2^T^, that were isolated from biotopes under strong geochemical and technogenic pressure (1). Here, we report the draft genome sequence of strain С2, the type strain of the genus.

Strain C2^T^ is a mesophilic and neutrophilic, non-methane-utilizing methylotroph with the serine pathway of C1 assimilation. Interestingly, this bacterium accumulates poly-3-hydroxybutyrate (PHB) with ultra-high molecular mass, which has not previously been shown for methylotrophs.

Genomic DNA was extracted from culture using a genome DNA kit (Sigma). DNA was sonicated (S2/E210 Focused-ultrasonicator, Covaris, USA), fractionated electrophoretically (E-Gel), and finally was used for 400-500 bp library preparation according to the manufacturer’s protocols (Thermo Fisher Scientific, Inc., USA).

The draft genome sequence of *M. halotolerans* С2^T^ was determined using the semiconductor genome analyzer Ion Torrent PGM (Thermo Fisher Scientific, Inc.) using a 400-bp sequencing kit and 318 v2 chip. The total 1,435,946 raw reads were assembled *de novo* in SPAdes version 3.7.1 (2), resulting in 26 contigs (115× coverage) with an average G+C content of 63.56%. The *N*_50_ was 388,255 bp, and the longest contig was 895,239 bp. The draft genome of *M. halotolerans* C2^T^ was found to comprise 3,191,628 bp and encode 3,175 predicted protein-coding genes (CDSs), with 46 tRNAs, one tmRNA, and three rRNAs. The CDSs, as well as the rRNA sequences, were predicted and annotated using Prokka version 1.11 (3) with the Barrnap version 0.5 plug-in (http://www.vicbioinformatics.com/software.barrnap.shtml).

The gene cluster *mxaFJGIRSACKLD*, encoding the methanol dehydrogenase, was identified. Genes *pqqABCDEF*, scattered across the genome, and *xoxFJJG* were predicted. The genes encoding enzymes involved in biosynthesis of the two pterins H_4_MPT and H_4_F, as well as the glutathione-dependent formaldehyde-activating enzyme, were identified.

Genes encoding key enzymes of the serine pathway were detected, and the presence of crotonyl-CoA carboxylase/reductase (1.3.1.85) confirms the implementation of an ethylmalonate cycle for glyoxylate regeneration.

Two paralogs of class I poly-beta-hydroxybutyrate polymerases (2.3.1.-) [intracellular PHB depolymerase *phaZ*, the acetyl-CoA acetyltransferase (*phbA*)], three acetoacetyl CoA reductases (*phbB*), and three phasins were identified.

The genetic potential for osmoregulation was confirmed by the presence of the gene cluster *ectABC-ask*, which is responsible for ectoine biosynthesis (4), and at least two trehalose biosynthesis pathways (*TreS* and *TreYZ*). Remarkably, upstream of the gene cluster of *ectABC-ask*, MarR proteins as putative transcriptional regulators were identified.

The genes encoding glutamate dehydrogenase, glutamate synthase, and glutamine synthetase were found, in agreement with enzyme assay data, which indicates that ammonia was assimilated through both the glutamate cycle and the reductive amination of α-ketoglutarate.

Genes encoding enzymes of the incomplete tricarboxylic acid cycle (devoid of α-ketoglutarate dehydrogenase and succinyl-CoA synthetase), nitrate metabolism, and nitrite metabolism were detected as well as the genes of siderophore (enterobactin) and polyketide (*pksJLM*) production. Key genes associated with the RuMP or the ribulose-bisphosphate-pathway of C1 assimilation were absent, in compliance with the enzyme assay data (1).

### Accession number(s).

This whole-genome shotgun project has been deposited in DDBJ/ENA/GenBank under the accession number MASI00000000. The version described in this paper is the first version, MASI01000000.
